# Supplementary material for: An oligogenic architecture underlying ecological and reproductive divergence in sympatric populations
Source: eLife. 2023 Feb 28;12:e82825. doi: 10.7554/eLife.82825 (PMC9977317; doi:10.7554/eLife.82825)
Supplement: Figure 5—figure supplement 1—source data 1. [file elife-82825-fig5-figsupp1-data1.docx]

| **Interacting QTL** | | |  | **Tests for epistatic interactions** | | | | | |
| --- | --- | --- | --- | --- | --- | --- | --- | --- | --- |
| Chr | Pos 1 (cM) | Pos 2 (cM) |  | LOD(Mf) | p(Mf) | LOD(Mf-M1) | p(Mf-M1) | LOD(Mf-Ma) | p(Mf-Ma) |
| 1:1 | 46 | 78 |  | **13.9** | **0** | **8.12** | **0.016** | 4.25 | 0.460 |
| 1:2 | 42 | 72 |  | **22.1** | **0** | **13.09** | **0.000** | **6.56** | **0.018** |
| 1:3 | 84 | 12 |  | **14.7** | **0** | **9.01** | **0.004** | 2.78 | 0.925 |
| 2:3 | 93 | 12 |  | **16.6** | **0** | **7.61** | **0.033** | 1.16 | 1.000 |
