# Supplementary material for: An oligogenic architecture underlying ecological and reproductive divergence in sympatric populations
Source: eLife. 2023 Feb 28;12:e82825. doi: 10.7554/eLife.82825 (PMC9977317; doi:10.7554/eLife.82825)
Supplement: Figure 5—figure supplement 1—source data 2. [file elife-82825-fig5-figsupp1-data2.docx]

| **Interacting QTL** | | |  | **Tests for additivity** | | | |
| --- | --- | --- | --- | --- | --- | --- | --- |
| Chr | Pos 1 (cM) | Pos 2 (cM) |  | LOD(Ma) | p(Ma) | LOD(Ma-M1) | p(Ma-M1) |
| 1:1 | 12 | 100 |  | **9.62** | **0** | **3.87** | **0.003** |
| 1:2 | 13 | 90 |  | **15.57** | **0** | **6.53** | **0.000** |
| 1:3 | 101 | 25 |  | **11.97** | **0** | **6.23** | **0.000** |
| 2:3 | 74 | 23 |  | **15.49** | **0** | **6.45** | **0.000** |
