# Supplementary figures and images for: An oligogenic architecture underlying ecological and reproductive divergence in sympatric populations
Source: eLife. 2023 Feb 28;12:e82825. doi: 10.7554/eLife.82825 (PMC9977317; doi:10.7554/eLife.82825)

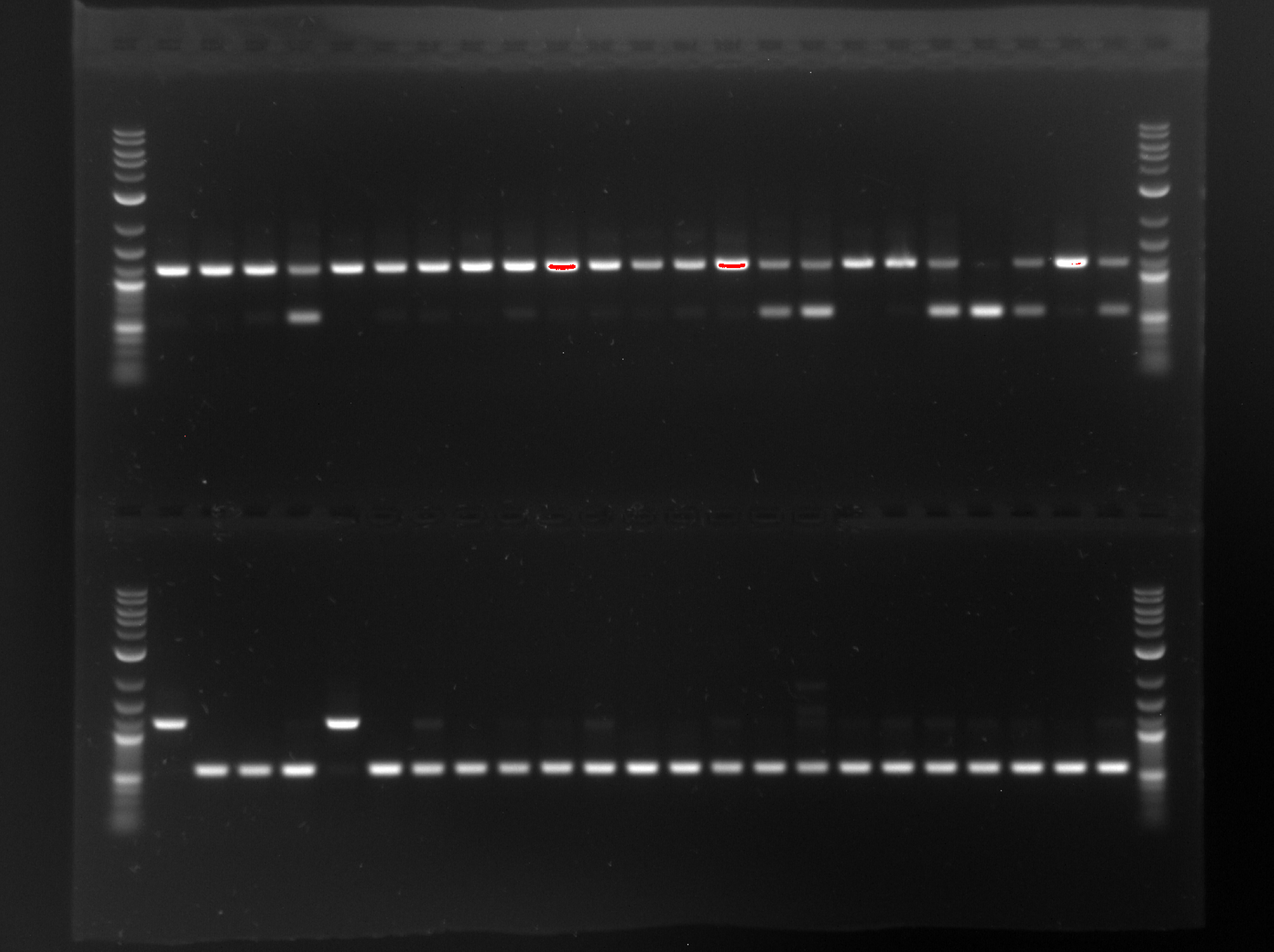

Supplement: Figure 5—figure supplement 4—source data 1. [file elife-82825-fig5-figsupp4-data1.zip › Fig5_S4_SourceData/Sina Schirmer 2018-05-17 12hr 44min.tif]

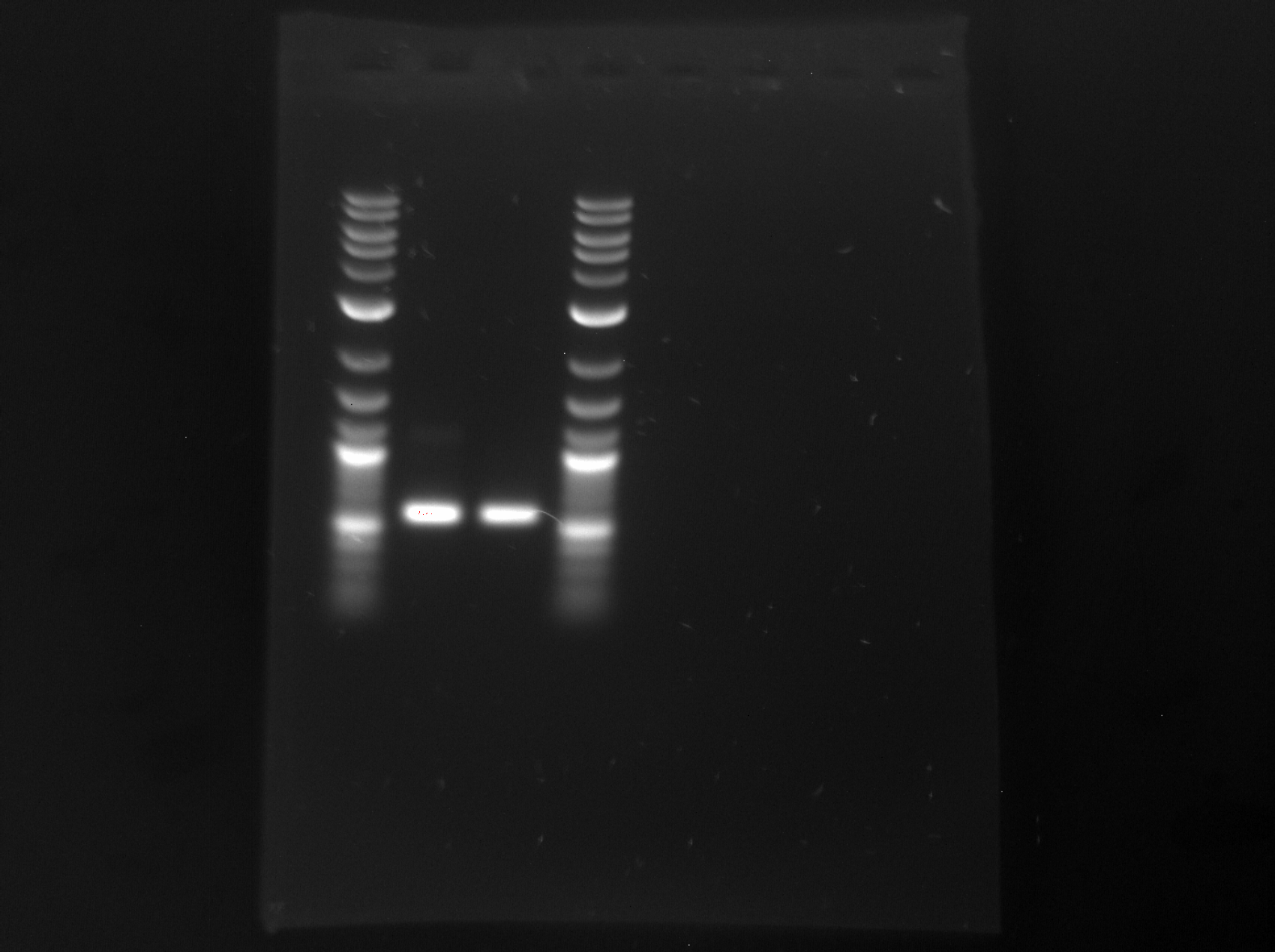

Supplement: Figure 5—figure supplement 4—source data 1. [file elife-82825-fig5-figsupp4-data1.zip › Fig5_S4_SourceData/Sina Schirmer 2018-05-17 12hr 51min.tif]

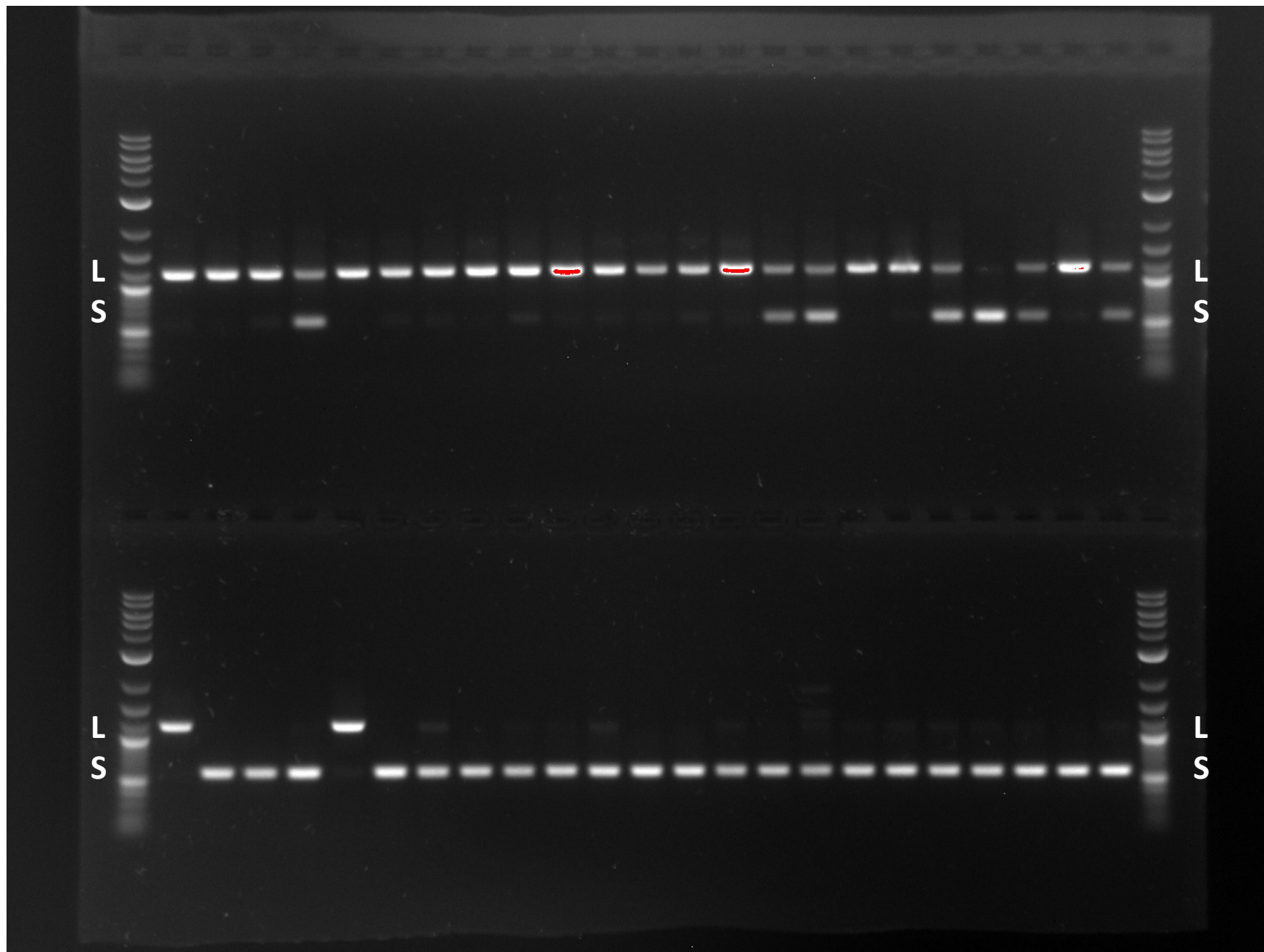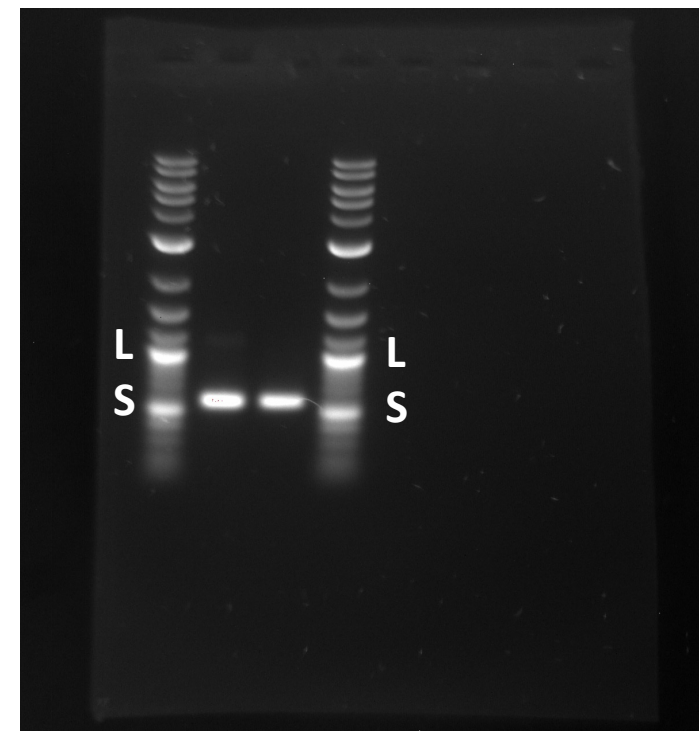

Supplement: Figure 5—figure supplement 4—source data 1. [file elife-82825-fig5-figsupp4-data1.zip › Fig5_S4_SourceData/BandsLabelled.pdf]
