# Supplementary material for: An oligogenic architecture underlying ecological and reproductive divergence in sympatric populations
Source: eLife. 2023 Feb 28;12:e82825. doi: 10.7554/eLife.82825 (PMC9977317; doi:10.7554/eLife.82825)
Supplement: Supplementary file 3. [file elife-82825-supp3.docx]

|  | Number of variants |
| --- | --- |
| Chr1 | 261.739 |
| In(1a), left arm | 15.972 |
| In(1b) | 83.456 |
| In(1a), right arm | 92.064 |
| In(1c), right arm | 52.125 |
| Chr2 | 249.948 |
| In(2L) | 114.556 |
| In(2R) | 77.001 |
| Chr3 | 191.892 |
| In(3L) | 53.786 |
| In(3R) | 51.788 |
